# Development of a δ^13^C and δ^34^S Isotope Analysis Method for Sulfadimidine and Its Potential to Trace Contaminant Transformation in Groundwater Systems

**DOI:** 10.1021/acs.analchem.4c05625

**Published:** 2025-02-14

**Authors:** Steffen Kümmel, Cecilie F. Ottosen, Mikael E. Olsson, Mette M. Broholm, Poul L. Bjerg, Hans H. Richnow

**Affiliations:** † Department of Technical Biogeochemistry, Helmholtz Centre for Environmental Research (UFZ), Permoserstraße 15, 04318 Leipzig, Germany; ‡ Department of Environmental and Resources Engineering, Technical University of Denmark (DTU), Bygningstorvet Building 115, 2800 Kgs Lyngby, Denmark; § Isodetect GmbH, Deutscher Platz 5b, 04103 Leipzig, Germany

## Abstract

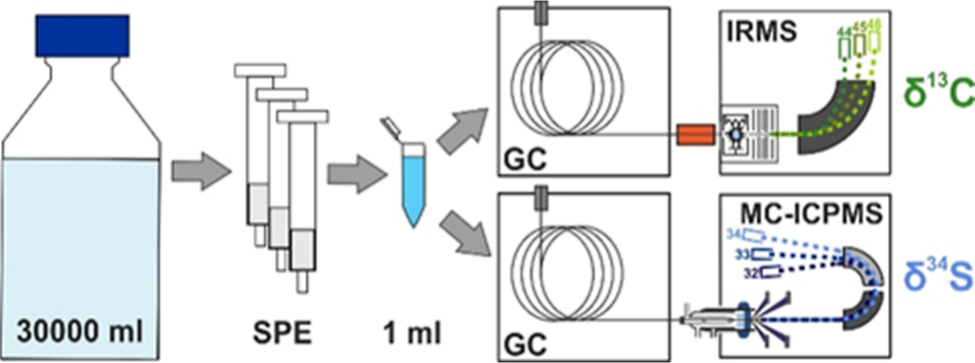

The widespread occurrence of micropollutants like the antibiotic sulfadimidine in the environment has become a growing concern. Compound-specific stable isotope analysis (CSIA) offers a powerful tool for tracking the fate of such pollutants, but its application is often limited by low sensitivity. To address this limitation, a large-scale solid-phase extraction method was developed to extract, enrich, and isolate sulfadimidine for δ^13^C- and δ^34^S-CSIA. Each step of the method was carefully evaluated, ensuring no detectable isotope artifacts. The limit of quantification was determined as 1.1 nmol of carbon and 1.2 nmol of sulfur directly injected on the column. Applied to groundwater samples from a contaminated site in Denmark, the method allowed for the analysis of concentrations as low as 0.17 mg/L, with a concentration factor of up to 10,000 used to enrich sulfadimidine. This is the first study to analyze δ^13^C and δ^34^S for sulfadimidine in aquifer water samples and highlights the potential of CSIA for tracking sulfadimidine transformations in contaminated water environments.

## Introduction

Sulfonamides are a major class of antibiotics and are considered emerging contaminants of concern due to their presence in the environment, which can contribute to the spreading of antibiotic resistance.^1^ Sulfonamides are present in multiple environmental compartments, yet aspects of the natural degradation processes influencing their fate remain to be explored.^2^ To study degradation processes, there is a need for methods to track and explore the transformation of all relevant sulfonamides under various environmental conditions. Currently, the primary focus in research has been to unravel the fate of sulfamethoxazole (SMX)—one of the most prevalent sulfonamides in the environment—yet other sulfonamides are also frequently detected and found at similar levels.^3,4^ SMX has a five-membered heterocyclic ring-substituent, while many of the common sulfonamides have six-membered heterocyclic ring-substituents,^2^ which is likely to influence fate patterns. Therefore, in this study we focused on sulfadimidine (4-amino-*N*-(4,6-dimethylpyrimidin-2-yl)benzenesulfonamide; C_12_H_14_N_4_O_2_S; also known as sulfamethazine) as a representative for a sulfonamide with a six-membered heterocyclic substituent, which is frequently detected in environmental samples.^3^

Assessing the concentration decrease of parent compounds alongside the detection of transformation products is frequently utilized to elucidate contaminant degradation.^5^ However, measuring the concentrations of parent compounds alone is often insufficient to differentiate among various transformation processes. Similarly, the detection of transformation products alone cannot provide a complete picture, as the same transformation products can arise from multiple, distinct degradation pathways. Therefore, this approach may be insufficient for an effective assessment of the level of contaminant degradation in the environment. In this context, compound-specific stable isotope analysis (CSIA) can serve as a complementary technique for monitoring contaminant degradation in environmental compartments.^6−8^ CSIA makes use of the different reaction kinetics of isotopologues (i.e., molecules that differ only in their isotopic composition) during bond transformations to analyze and characterize the transition changes occurring in (bio)chemical reactions. Thereby, the kinetic isotope effect (KIE) provides insights into the transition state of the first irreversible reaction step that initiates the transformation of a chemical by using the isotopic composition of the parent compound as an indicator in a quasi-closed system. A normal KIE arises from the preferential reaction of the lighter isotopes (e.g., ^12^C) over heavier ones (e.g., ^13^C) due to slight differences in the activation energies. Hence, heavier isotopes are usually more enriched in the residual fraction of the reactant and less abundant in the product fraction, which can be quantified using the Rayleigh equation.^9^ Accordingly, alterations in the isotopic composition of a certain compound can thus serve as a proxy for the first step of a degradation reaction and can be utilized to track transformation pathways and extents of contaminant degradation.^10−12^ For a better understanding of degradation mechanisms of organic compounds, simultaneous quantification of isotopic compositions from two or more stable isotopes can be achieved through multi-element CSIA (ME-CSIA). The correlation of multi-isotope effects may enable the characterization of the molecular entity involved in the first irreversible reaction while negating rate limitations on reaction steps that occur prior to the bond cleavage.^12,13^

Previously, Birkigt and colleagues^14^ employed a high performance liquid chromatography (HPLC) IRMS method to assess the carbon isotopic composition of SMX. However, the complexity and limited ability for intricate chromatographic separation of this method render it unsuitable for analyzing environmental samples, which typically have high matrix contents. Moreover, HPLC-IRMS is limited to the analysis of stable carbon isotopic compositions and, in consequence, cannot be used to assess the stable isotopic compositions of other elements such as hydrogen, nitrogen, and sulfur. To address these limitations, Ouyang and colleagues^15^ devised a gas chromatographic (GC) method for SMX analysis, eliminating the necessity for an additional derivatization and thus preventing potential bias in isotope ratios. Thereby, a foundation for isotope analysis of sulfonamides in environmental samples was built. Liu and colleagues^16^ further improved this method by applying it to develop multi-isotope concepts for the analysis of SMX (^2^H, ^13^C, ^15^N ^33^S, and ^34^S), enabling the characterization of the direct phototransformation of SMX under a typical sunlight spectrum.^16^

Analyzing environmental samples presents additional challenges, particularly due to low concentrations and matrix interferences.^17^ Given that CSIA is a relatively insensitive method, the implementation of robust extraction and cleanup procedures is necessary.^8^ However, a prerequisite for these procedures is that they must effectively isolate the respective target compound and should be executed with meticulous precision to avoid the introduction of isotope artifacts, which may compromise the analytical accuracy. Various methods have been employed to extract and concentrate contaminants from environmental samples for CSIA,^18,19^ including, e.g., liquid–liquid extraction, liquid–solid extraction, and Soxhlet extraction. However, for large-volume environmental water samples, solid-phase extraction (SPE) methods could be employed due to their effectiveness in extracting and concentrating contaminants.^20,21^

Accordingly, the objectives of this study were to (1) develop and validate a robust extraction, enrichment, and isolation approach for sulfadimidine that is compatible for concentration levels typically observed in groundwater samples, (2) adapt GC methods for precise analysis of δ^13^C and δ^34^S of sulfadimidine, and (3) apply these validated approaches to groundwater samples from a contaminant plume with sulfadimidine and other sulfonamides. To the best of our knowledge, there are no established methods for δ^13^C and δ^34^S analyses of this compound available, and our study represents the first application of CSIA for sulfadimidine in groundwater samples.

## Experimental Section

### Chemicals

The sulfadimidine (VETRANAL grade) used for developing the SPE method in the laboratories of the Technical University of Denmark (DTU) was obtained from Merck (Germany). The sulfadimidine (VETRANAL grade) used for method development related to stable isotope analyses at the Laboratories for Stable Isotopes at the Helmholtz-Centre for Environmental Research, Leipzig (UFZ) was obtained from Sigma-Aldrich (Germany). Methanol, acetonitrile, formic acid (LCMS grade), and acetone (GCMS grade) were purchased from Merck (Germany). The sulfadimidine-d4 used as an internal standard was purchased from LGC Standards (Germany).

### Concentration Analysis

The sulfadimidine concentrations were analyzed using HPLC-triple quadrupole mass spectrometry (1290 Infinity II- 6470 LC-QQQ, Agilent Technologies, USA). For instrument calibration, individual stock solutions of sulfadimidine and sulfadimidine-d4 were initially prepared at a concentration of 1 g/L by dissolving 5 mg of the substance in 5 mL of methanol. These stock solutions were then used to create working standards ranging from 0.02 to 20 μg/L sulfadimidine in Milli-Q grade water.

Prior to analysis, extracted samples were diluted 10,000 times in Milli-Q grade water, and sulfadimidine-d4 was added as an internal standard, resulting in a final concentration of 10 μg/L. Samples not prepared by SPE were analyzed undiluted but with the same concentration of the internal standard. Chromatographic separation was performed using a 2.1 mm × 100 mm, 3 μm, 120 Å reversed-phase column (PolarAdvantage II C18 PA2, Thermo Scientific, Germany). The aqueous phase (A) consisted of 0.1% formic acid in Milli-Q-grade water, while the mobile phase (B) was acetonitrile with 0.1% formic acid. The following gradient was applied: 0% B (initial), 0–5% B (2–6 min), 5% B (6–15 min), and 5–85% B (15–18 min) at a flow rate of 0.5 mL/min. The column compartment was maintained at 35 °C. Column regeneration time was 3 min under the initial conditions (100% A). A 10 μL sample was injected, and the retention time for sulfadimidine was 9.7 min. The detection was performed using a dynamic multiple reaction monitoring (dMRM) with the protonated molecular ion [M + H]^+^ as the precursor and the most predominant fragment ion as the product ion (sulfadimidine: *m*/*z* = 279 → 156; sulfadimidine-d4: *m*/*z* = 283 → 160). Positive electrospray ionization was employed with a source temperature of 350 °C, a nebulizer gas flow of 11 L/min at 40 psi, and a sheath gas at 400 °C with a flow rate of 12 L/min. The collision-induced dissociation voltage was set to 10 V, the fragmentor voltage to 100 V, and the cell acceleration voltage to 7 V.

### Solid-Phase Extraction

The SPE method used in the present work is based on the protocol described by Diaz-Cruz and colleagues^22^ but has been adapted to extract a 3000 mL sample per cartridge with additional cleanup steps for final extract purification. Prior to SPE, each 30 L sample was divided into ten fractions. Each fraction underwent SPE using a 24-position vacuum manifold (Chromabond SPE, Macherey-Nagel, Germany) with a connected vacuum pump (Laboport N820G, KNF Neuberger, Germany). Each fraction (3000 mL) was extracted using one 2 g/20 mL Oasis HLB column (Waters, USA). The cartridges were conditioned sequentially with 20 mL of methanol containing 50 mM formic acid, 20 mL of acetone containing 50 mM formic acid, and 10 mL of methanol. The sample was loaded at a flow rate of 10 mL/min, followed by a washing step with 200 mL of methanol. Subsequently, the samples were eluted with 12 mL of methanol containing 50 mM formic acid, followed by 12 mL of acetone containing 50 mM formic acid, and both fractions were combined. The resulting eluate was dried under a gentle nitrogen stream until approximately 2 mL of eluate remained. Finally, the eluates from the ten cartridges were combined, lyophilized, and reconstituted in 1 mL of acetone. After centrifugation at 3,000*g* for 5 min, the supernatant was transferred and used for further analysis.

### Analysis of Sulfadimidine via Gas Chromatography

Prior to isotope ratio measurements, a GC method was developed for sulfadimidine using an Agilent 7890 A GC system (Agilent Technologies, USA) equipped with a 5975C mass spectrometer (MS; Agilent Technologies, USA) and a CombiPAL autosampler (CTC Analytics AG, Switzerland). Chromatographic separation employed a Zebron ZB-1 column (60 m × 0.32 mm × 0.25 μm; Phenomenex, Germany) with a constant helium carrier gas flow rate of 2.0 mL/min. The temperature program consisted of an initial hold at 40 °C for 5 min, followed by an increase of 10 °C/min to 320 °C, and a subsequent hold at 320 °C for 15 min. Ionization was performed in electron impact mode, and the transfer line, ion source, and quadrupole temperatures were 280, 230, and 150 °C, respectively. To assess the impact of the injector temperature on sulfadimidine stability, temperature variations were tested ranging from 180 to 280 °C in 20 °C increments. Samples were injected in split mode (at ratios of 1:5 and 1:10) with injection volumes of 1 μL.

### Analysis of Stable Carbon Isotope Ratios (δ^13^C) of Sulfadimidine

Compound-specific stable carbon isotope analysis of sulfadimidine was performed on a Thermo Scientific MAT 253 isotope-ratio mass spectrometer (IRMS; Thermo Fisher, Germany) interfaced with an Agilent 7890 A GC system (Agilent Technologies, USA) via a GC-IsoLink and a ConFlo IV interface (Thermo Fisher, Germany). Samples were injected via a CombiPAL autosampler (CTC Analytics AG, Switzerland) into the split/splitless injector of the GC system with injection volumes adjusted to 1–5 μL depending on sulfadimidine concentrations in the samples. Separation of samples was conducted on a Zebron ZB-1 column employing the same temperature program as that utilized for GC–MS analysis (described above). For carbon isotope analysis, sulfadimidine was converted to CO_2_ at 1000 °C in the combustion oven of the GC-IsoLink equipped with a commercially available oxidation reactor (1040920, Thermo Fisher, Germany). All samples were analyzed in triplicates. The total uncertainty of δ^13^C measurements was ±0.5‰ incorporating both accuracy and reproducibility.^6,23^

Bulk stable carbon isotope analysis was carried out using an EuroEA3000 elemental analyzer (EA, HEKAtech, Germany) linked to a Thermo Scientific MAT 253 IRMS (Thermo Fisher, Germany) interfaced via a ConFlo IV (Thermo Fisher, Germany) following previously established protocols.^24^ Normalization of the δ^13^C raw data was carried out by a two-point calibration employing reference materials (IAEA-CH_6_ = −10.45 ± 0.04‰ and IAEA-CH_7_ = −32.15 ± 0.05‰; for confirmation, IAEA-CH_3_ = −24.72 ± 0.04‰ was used) from the International Atomic Energy Agency (Vienna, Austria). The precision was always better than ±0.1‰.

### Analysis of Stable Sulfur Isotope Ratios (δ^34^S) of Sulfadimidine

Compound-specific stable sulfur isotope analysis of sulfadimidine was analyzed using a GC (Trace 1310, Thermo Fisher, Germany) coupled with multiple-collector inductively coupled plasma MS (GC-MC-ICP-MS, Neptune, Thermo Fisher, Germany), as recently described elsewhere.^25^ Samples were injected via a TriPlus RSH autosampler (Thermo Fisher, Germany) into the split/splitless injector of the GC system with injection volumes adjusted to 1–5 μL depending on sulfadimidine concentrations in the samples. Separation of samples was conducted on a Zebron ZB-1 column employing the same temperature program as utilized for GC–MS analysis (described above). All samples were analyzed in triplicates. The total uncertainty of δ^34^S measurements was ±0.3‰ incorporating both accuracy and reproducibility. The δ^34^S values were calibrated to the SMOC scale by applying a two-point calibration approach. The calibration was performed using in-house standards with attested off-line sulfur isotopic compositions, including thiophene (THI, δ^34^S = −1.74 ± 0.18‰) and dimethyl disulfide (DMDS, δ^34^S = 8.60 ± 0.17‰). In addition, sulfur hexafluoride (SF_6_, δ^34^S = −1.16 ± 0.15‰) and diethyl sulfide (DES, δ^34^S = 6.36 ± 0.20‰) were used for validating the calibration, as described elsewhere.^25^

Bulk stable sulfur isotope analysis was carried out using an EuroEA3000 EA (HEKAtech, Germany) linked to a MC-ICP-MS (Neptune, Thermo Fisher, Germany), as recently described elsewhere.^26^ Normalization of the δ^34^S raw data was carried out by a two-point calibration employing reference materials (IAEA-S-2 = 22.62 ± 0.16‰ and IAEA-S-3 = −32.49 ± 0.16‰, for confirmation, IAEA-S-1 = −0.30‰ was used) from the International Atomic Energy Agency (Vienna, Austria). The precision was always better than ±0.3‰.

### Isotope Data Analysis

Isotope ratios were reported in delta notation (δ^13^C and δ^34^S) based on eq 1
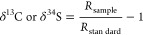
1*R*_sample_ and *R*_standard_ represent the ratios of ^13^C/^12^C or ^34^S/^32^S of the sample and the international standard, respectively. The international standard for carbon isotopes is Vienna Pee Dee Belemnite (VPDB), and the one for sulfur isotopes is Vienna Cañon Diablo meteorite (VCDT). The values are denoted in delta notations as outlined by Brand and Coplen.^27^

For dual isotope analysis, the lambda value Λ was calculated using the regression method. Accordingly, the Λ_S/C_ value was obtained by calculating the slope of the fitted line, where the Δ^34^S values were plotted against the Δ^13^C values. Additionally, the 95% confidence level of the slope was computed.

### Field Site and Sample Collection

Environmental samples were collected from an anaerobic sandy aquifer contaminated by a sulfonamide plume originating from a former pharmaceutical production facility, as previously described.^28^ In this plume, sulfadimidine was recently found at concentrations up to 530 μg/L. The sulfonamide plume discharges to a stream approximately 1.5 km downgradient of the factory site.^29^

Five wells (GR1-GR5) were selected for sampling in order to obtain samples from different parts of the plume with sufficient concentration levels for isotope analysis. These sampling points are placed along the main flowline in the following order: GR1-GR4, whereas GR5 is slightly outside the flowline (Figure S1). The water samples were collected in 5 L plastic containers, and 30 L of water was collected at each sampling point. The samples were stored cold at 10 °C prior to SPE.

## Results and Discussion

### Development of a GC Method for Sulfadimidine

**Figure 1:**
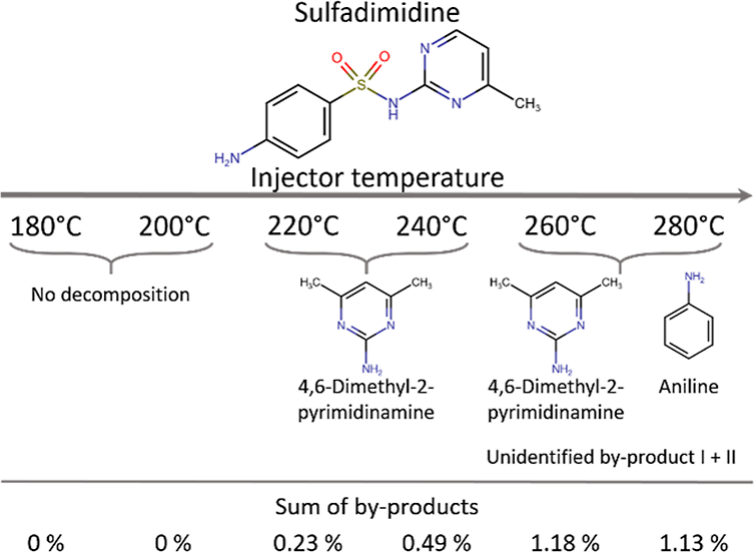
Proposed structures of byproducts resulting from thermal decomposition of sulfadimidine identified via GC–MS.

A GC–MS method for sulfadimidine was developed based on the report of Ouyang and colleagues.^15^ The initial stage involved optimizing the injector temperature. To accomplish this, a new, clean, and deactivated liner (SGE inlet liner 4 mm ID Tapper QW PK5, Trajan Scientific, Australia) was employed, and the injector temperature was varied from 180 to 280 °C to ensure efficient vaporization while preventing sulfadimidine decomposition. Identification of the sulfadimidine peak and its decomposition products was achieved by comparing them with entries in the NIST library with a confidence level exceeding 90%. Certainly, the peak area of sulfadimidine exhibited variation with changing injector temperature (Figure S2), with the highest peak area recorded at an injector temperature of 200 °C. At a temperature of 180 °C, the peak area was notably reduced and accompanied by a larger standard deviation, likely resulting from a less reproducible transfer of sulfadimidine to the GC-column due to inconsistent vaporization. Further increasing the injector temperature to >200 °C did not result in higher peak areas. Instead, byproducts began to form, likely stemming from sulfonamide decomposition within the injector and consequently causing the observed reduction in peak area (Figure S2). A maximum of four distinct byproducts resulting from the thermal decomposition of sulfadimidine were detected. Among these, only two could be tentatively identified through comparison with the NIST library: 4,6-dimethyl-2-pyrimidinamine, which became apparent at injector temperatures of 220 °C and above, and aniline, which was detected at injector temperatures of 260 and 280 °C (Figure 1). The other unidentified byproducts were solely observed at injector temperatures of 260 and 280 °C.

The combined peak areas of the byproducts relative to the sum of the peak areas of all detected peaks in each sample (including the sulfadimidine peak) ranged up to a maximum of 1.2%. The proportion of byproduct peak areas increased with higher injector temperatures. However, once reaching 260 °C, the overall amount of byproducts stabilized and did not exhibit further increases even at temperatures of 280 °C (Tables S1 and S2). As anticipated, the formation of byproducts was lower at a split ratio of 1:10 compared to 1:5 at the same injector temperature. This difference can be attributed to the fact that with a split ratio of 1:10, only about half of the sample is introduced into the injector, whereas at a split ratio of 1:5, a larger portion enters. Consequently, the lower initial concentration of the analyte entering the analytical system reduces the likelihood of sulfadimidine decomposition, which is most likely to occur on the inner wall of the liner. Unlike the study conducted by Ouyang and colleagues,^15^ which examined SMX, we noted a comparatively minor generation of byproducts. Additionally, this formation commenced at higher injector temperatures, indicating a higher thermal stability of sulfadimidine (first mass loss at 234 °C^30^), compared to SMX (first mass loss at 205 °C^31^). However, since no decomposition of sulfadimidine was detected at 200 °C (regardless of whether the split ratio was 1:5 or 1:10), and concurrently, the peak area was at its maximum (Figure S2), 200 °C was chosen as the optimal injector temperature for sulfadimidine analysis via GC. At this specified injector temperature, sulfadimidine standards with concentrations ranging from 1 to 100 mM were examined by GC–MS. Irrespective of the split ratio employed (either 1:5 or 1:10), all samples exhibited a consistent linear response, as indicated by the coefficients of determination (*R*^2^) derived from linear regression analysis of the data (Figure S3). However, it should be mentioned that at sulfadimidine concentrations of 100 mM and with a split ratio of 1:5, a total of 1.5% byproducts were generated. Yet, these small quantities, likely resulting from sulfadimidine decomposition in the injector, did not cause a discernible decrease in the sulfadimidine peak area.

### Dependency of Isotope Values on the Amount of Sulfadimidine

**Figure 2:**
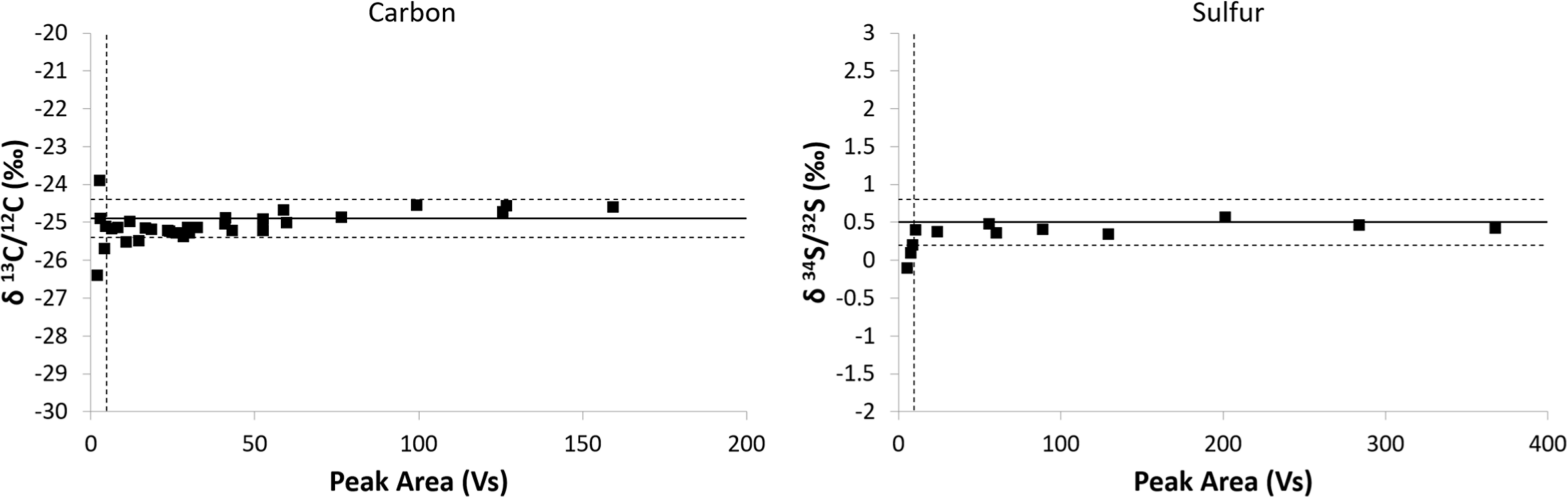
Carbon (left panel) and sulfur (right panel) stable isotope ratios of sulfadimidine across different concentrations. Solid horizontal line represents the mean value derived from elemental isotope analysis (δ^13^C = −24.9 ± 0.1‰ or δ^34^S = 0.5 ± 0.2‰), whereas dashed horizontal lines depict the typical uncertainties of GC-IRMS or GC-MC-ICP-MS (δ^13^C = ±0.5‰ or δ^34^S = 0.3‰). Dashed vertical lines indicate the minimum peak areas required for reaching the linearity ranges of each element.

The relationship between the concentration of sulfadimidine and its isotopic composition was examined to determine the dynamic range of concentrations (linear range), where the isotopic composition can be reliably analyzed. Hence, carbon (δ^13^C) and sulfur (δ^34^S) isotope signatures of sulfadimidine standards ranging from 0.5 to 100 mM were evaluated by GC-IRMS or GC-MC-ICP-MS employing the optimal GC operational parameters outlined above. Subsequently, peak areas of sulfadimidine were plotted against the isotopic compositions (Figure 2).

This experiment demonstrated that carbon and sulfur isotope ratios stabilized within a consistent range, yielding reproducible results of δ^13^C = −25.1 ± 0.3‰ and δ^34^S = 0.4 ± 0.1‰ when the peak areas were above 5 Vs for carbon and 10 Vs for sulfur, respectively (Figure 2, dashed vertical lines). This corresponds to a limit of quantification of approximately 1.1 nmol of carbon and 1.2 nmol of sulfur directly injected on column, which aligns with recent findings for SMX.^16^ Accordingly, these results indicate that the isotope ratios remained unaffected by variations in the bulk concentrations of sulfadimidine provided that the signal sizes were sufficiently high. Moreover, the bulk δ^13^C and δ^34^S values of the sulfadimidine standard utilized for this experiment were determined to be −24.9 ± 0.1‰ and 0.5 ± 0.2‰ through elemental analysis (Figure 2, solid horizontal lines). Consequently, the isotope ratios obtained from GC-IRMS or GC-MC-ICP-MS coincide with the bulk value obtained directly from elemental analysis, validating the GC-based methods for sulfadimidine analysis.

### Large Scale SPE Sample Purification and Concentration

To exclude that isotope fractionation was introduced by the SPE procedure when processing a sample, extraction tests were conducted using a sulfadimidine standard at elevated concentrations (i.e., 1 and 10 mM). These extractions resulted in concentration factors of approximately 630- and 970-fold, respectively. The accuracy of the entire SPE-CSIA method, as assessed in these extraction tests, showed a maximum deviation of ±0.2‰ for carbon and 0.1‰ for sulfur (Table 1). Considering the analytical uncertainty for δ^13^C (±0.5‰) and δ^34^S (±0.3‰), all observed shifts can be regarded as negligible, and the SPE-procedure is applicable for subsequent isotope analysis.

**Table 1:** Effect of the SPE Procedure on the Isotopic Composition of Sulfadimidine

sample	δ^13^C (‰)	Δδ^13^C (‰)	δ^34^S (‰)	Δδ^34^S (‰)
sulfadimidine standard before SPE	–23.5 ± 0.1		0.1 ± 0.2	
sulfadimidine after SPE (10 mM)	–23.3 ± 0.2	0.2	0.0 ± 0.1	0.1
sulfadimidine after SPE (1 mM)	–23.3 ± 0.2	0.2	0.1 ± 0.2	0.0

### Determination of Isotope Values of Sulfadimidine in Groundwater Samples

In the last step, as a proof of concept, real environmental samples from a sulfonamide plume were processed by using the described workflow. Therefore, groundwater samples from an aquifer contaminated by a sulfonamide plume were collected, and sulfadimidine was extracted and concentrated via SPE. The concentrations of sulfadimidine in the groundwater samples prior to the SPE-treatment ranged from 171 to 364 μg/L (Table 2). The extraction procedure achieved concentration factors exceeding 10,000-fold, yielding a maximum concentration in the extracts of around 3000 mg/L (Figures S4 and S5).

**Table 2:** Sulfadimidine Levels in Groundwater Samples Prior to SPE Treatment as Well as Obtained Concentration Factors, Final Sulfadimidine Concentrations in the Extracts, and the Determined δ^13^C and δ^34^S Values of Sulfadimidine

well name	sulfadimidine in groundwater sample		concentration factor	sulfadimidine in extracts		δ^13^C (‰)	δ^34^S (‰)
	(μg/L)	(mM)		(mg/L)	(mM)		
GR1	258.5	0.9	≈7000	1796.2	6.5	–25.0 ± 0.2	1.9 ± 0.2
GR2	364.0	1.3	≈6200	2262.7	8.1	–24.7 ± 0.2	1.8 ± 0.2
GR3	297.0	1.1	≈5700	1682.6	6.0	–25.1 ± 0.2	2.0 ± 0.1
GR4	283.0	1.0	≈10,300	2921.2	10.5	–25.2 ± 0.1	2.1 ± 0.2
GR5	171.0	0.6	≈2200	367.5	1.3	–24.4 ± 0.3	1.7 ± 0.2

Subsequently, stable carbon and sulfur isotope ratios of sulfadimidine were analyzed, resulting in δ^13^C values ranging between −25.2 to −24.4‰ and δ^34^S values ranging between 2.1 and 1.7‰ (Table 2). Consequently, a maximal difference of 0.8‰ for carbon and 0.4‰ for sulfur was observed. Although these differences exceeded the standard deviations of the respective methods (i.e., 0.5‰ for carbon and 0.3‰ for sulfur), they exhibited only a weak enrichment compared to the recommended criterion, which should be more than 2‰ for carbon,^6^ for example.

However, no significant correlation was observed between the sulfadimidine concentration and the δ^13^C or δ^34^S values, respectively. Nonetheless, previous studies have documented normal carbon isotope effects during the microbial transformation of SMX under both aerobic (carbon isotopic fractionation = −0.6 ± 0.1‰) and anaerobic (carbon isotopic fractionation = −5.8 ± 0.7‰) conditions.^14,15^ These observations suggest that if microbial degradation is occurring at the investigated field site, a normal carbon fractionation for sulfadimidine would be expected in the aquifer.

**Figure 3:**
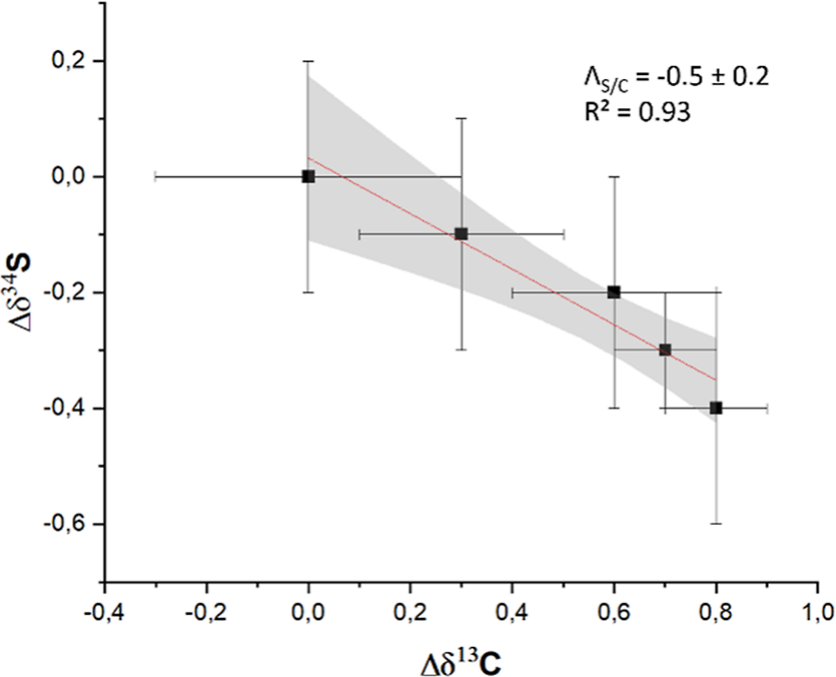
Dual-element plot (Λ_S/C_) of sulfadimidine detected in environmental samples collected from an anoxic sandy aquifer contaminated by a sulfonamide plume.

Finally, changes in carbon and sulfur isotope ratios were correlated, resulting in an Λ_S/C_ value of −0.5 ± 0.2 (Figure 3). However, as this is the first study on dual-element isotope fractionation of sulfadimidine employing carbon and sulfur isotope ratios, no lambda value is available in the literature for comparison. The only available lambda values for comparison are from photolysis experiments on SMX, which yielded Λ_S/C_ values of 1.8 ± 0.3 at pH7 or 2.1 ± 0.4 at pH3.^16^ These values are significantly higher than those determined for sulfadimidine in the aquifer, which is expected given that photolysis is not a relevant process in groundwater. It is important to note that the differences in isotope ratios observed in this study for both carbon and sulfur fall within two standard deviations (2σ) of the analytical uncertainty. Therefore, the observed correlation and the resulting Λ_S/C_ value should be interpreted cautiously. While the data may indicate the possibility of a C–S bond cleavage during the transformation of sulfadimidine, other physical processes, such as sorption or diffusion, could also contribute to the observed isotope effects. This highlights the need for further investigations to better understand the mechanisms driving the isotope fractionation of sulfadimidine under environmental conditions.

Overall, this experiment demonstrated that the developed workflow can be used for analyzing δ^13^C and δ^34^S values of sulfadimidine in real environmental samples and, moreover, confirmed the feasibility of dual-element analysis for assessing the attenuation of sulfadimidine in the environment and in groundwater in particular.

## Conclusions

In this study, a novel method for carbon and sulfur isotope analysis of sulfadimidine was successfully developed, comprising a comprehensive approach for the extraction, concentration, and cleanup of target analytes through large-volume SPE prior to isotope analysis. This method demonstrated both accuracy and precision in the isotope measurements, proving effective for sulfadimidine analysis in real field samples. Moreover, the concentration factors upon extraction and enrichment meet the concentration for multi-isotope analysis,^16^ and the successful application of this method in the experimental phase highlights its potential for broader applications in studying the fate of sulfadimidine and other sulfonamides using CSIA.

The method offers significant promise for future research, particularly in understanding the environmental behavior and transformation processes of sulfonamides. An intriguing aspect for further investigation is whether the minor changes in isotope fractionation observed in the field samples can be attributed to low enrichment factors or to minimal degradation of the parent compound. This consideration also applies to the analysis of degradation products, which may indicate either limited degradation of the parent compound or additional degradation of the products themselves.

Future work should focus on refining and expanding this methodology to improve its application and reliability. This includes broadening the range of analytes and testing across diverse environmental matrices to evaluate the method’s wider applicability. Moreover, a deeper exploration of the factors affecting isotope fractionation and degradation processes will be essential for improving the interpretation of isotope data. Our method development will contribute to a more thorough understanding of sulfonamide dynamics in environmental contexts, particularly groundwater systems, and enhance the effectiveness of CSIA for environmental monitoring and research.

## Supporting Information Available

Map of the field site showing the plume of a former pharmaceutical production facility; vaporization and decomposition effects of sulfadimidine standards at varying GC-injector temperatures and peak areas of sulfadimidine at various concentrations; GC–MS signals from a sulfadimidine standard and related byproducts formed due to thermal decomposition at varying GC split ratios; and GC-IRMS and HPLC-MS/MS chromatograms, demonstrating the reduction of matrix-induced signals as well as the concentration effect of the SPE treatment (PDF)
