# Supplementary material for: Development of a δ13C and δ34S Isotope Analysis Method for Sulfadimidine and Its Potential to Trace Contaminant Transformation in Groundwater Systems
Source: Anal Chem. 2025 Feb 14;97(7):4014–20. doi: 10.1021/acs.analchem.4c05625 (PMC11866275; doi:10.1021/acs.analchem.4c05625)
Supplement: Supplementary file 1 — ac4c05625_si_001.pdf [file ac4c05625_si_001.pdf]

Supporting Information

Development of a $\delta^{13}C$ and $\delta^{34}S$ isotope analysis method for sulfadimidine and its potential to trace contaminant transformation in groundwater systems

Steffen Kümmel[1,*], Cecilie F. Ottosen[2], Mikael E. Olsson[2], Mette M. Broholm[2], Poul L. Bjerg[2], Hans H. Richnow[1,3]

[1] Department of Technical Biogeochemistry, Helmholtz Centre for Environmental Research (UFZ), Permoserstraße 15, 04318 Leipzig, Germany

[2] Department of Environmental and Resources Engineering, Technical University of Denmark (DTU), Bygningstorvet building 115, 2800 Kgs. Lyngby, Denmark

[3] Isodetect GmbH, Deutscher Platz 5b, 04103 Leipzig, Germany

*Corresponding author (steffen.kuemmel@ufz.de)

Table of contents:

Figure S1. Map of the field site of a former pharmaceutical production facility S2

Figure S2. Vaporization and decomposition effects of a sulfadimidine standard at varying GC injector temperatures S3

Table S1. GC-MS signals from a sulfadimidine standard and related by-products due to thermal decomposition (split ratio 1:5) S4

Table S2. GC-MS signals from a sulfadimidine standard and related by-products due to thermal decomposition (split ratio 1:10) S5

Figure S3. Peak areas of sulfadimidine at various concentrations on GC-MS S6

Figure S4: Comparison of GC-IRMS chromatograms illustrating the effectiveness of the SPE method in reducing matrix interferences and concentrating sulfadimidine S7

Figure S5: Comparison of HPLC–MS/MS chromatograms illustrating the effectiveness of the SPE method in concentrating sulfadimidine S9

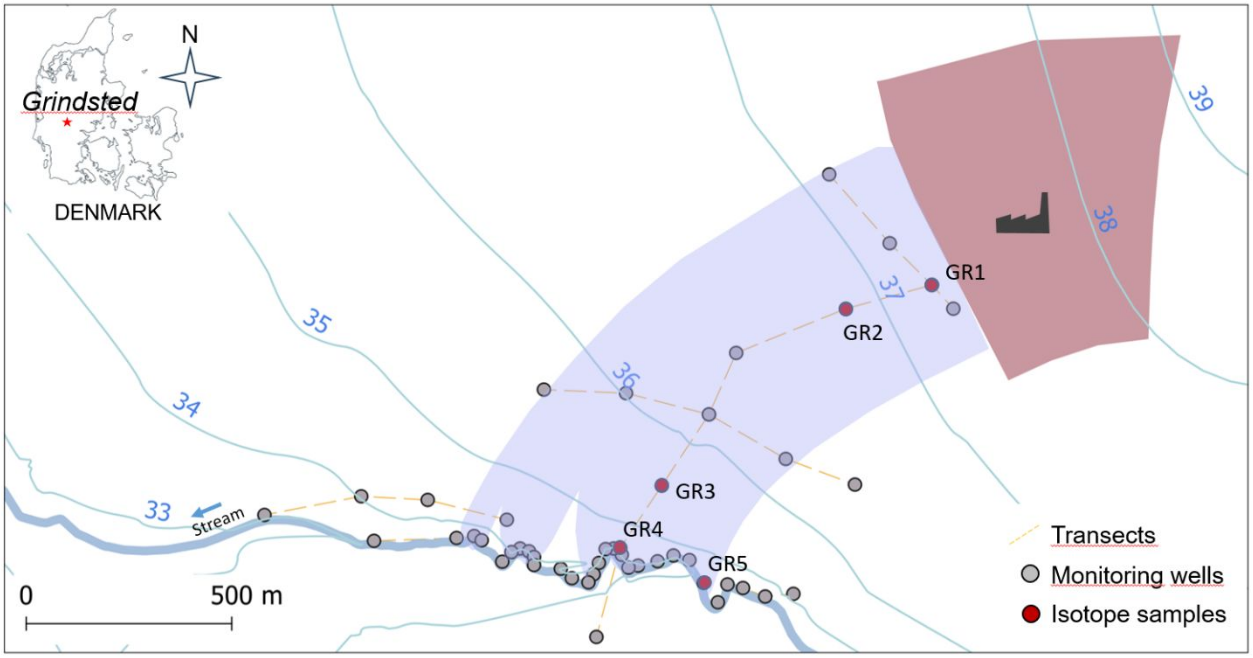

Figure S1: Map of field site showing the factory site (red area), the conceptual interpretation of the sulfonamide plume (purple area), the groundwater equipotential lines (light blue) and the stream (bold blue line).

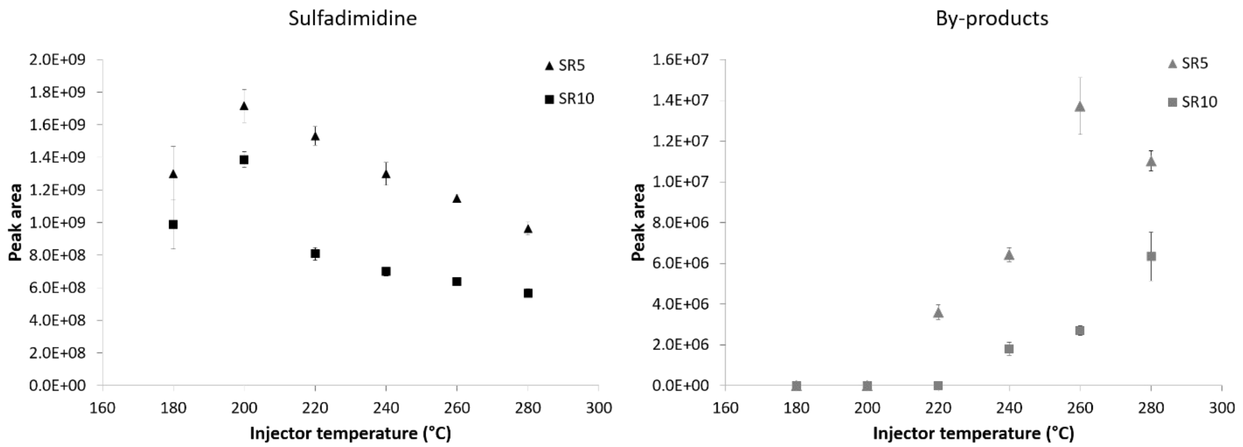

Figure S2: Vaporization and decomposition of a 50 mM sulfadimidine standard were examined at injector temperatures ranging from 180 °C to 280 °C in 20 °C increments. In the left panel, the peak area of sulfadimidine is depicted across different injector temperatures. The right panel illustrates the sum of the peak areas of the by-products resulting from the decomposition of sulfadimidine within the injector. Triangles represent sulfadimidine standards injected at a split ratio of 1:5, whereas squares correspond to standards injected at a split ratio of 1:10.

Table S1: GC-MS signals from a 50 mM sulfadimidine standard and the related by-products resulting from the thermal decomposition of sulfadimidine within the GC injector, set at a split ratio of 1:5.

Injector temp. (°C)	Compound	Average peak area	Relative proportion (%)	Sum of by-products (%)
180	Sulfadimidine	$1.30 \times 10^9 \pm 1.66 \times 10^8$	100.00	
200	Sulfadimidine	$1.72 \times 10^9 \pm 1.02 \times 10^8$	100.00	
220	Sulfadimidine	$1.53 \times 10^9 \pm 5.66 \times 10^7$	99.77	0.23
	4,6-Dimethyl-2-pyrimidinamine	$3.60 \times 10^6 \pm 3.58 \times 10^5$	0.23	
240	Sulfadimidine	$1.30 \times 10^9 \pm 7.06 \times 10^7$	99.51	0.49
	4,6-Dimethyl-2-pyrimidinamine	$6.43 \times 10^6 \pm 3.43 \times 10^5$	0.49	
260	Sulfadimidine	$1.15 \times 10^9 \pm 2.70 \times 10^7$	98.82	1.18
	4,6-dimethyl-2-pyrimidinamine	$8.83 \times 10^6 \pm 5.72 \times 10^5$	0.76	
	Aniline	$2.29 \times 10^6 \pm 2.68 \times 10^5$	0.20	
	Unidentified by-product I	$1.65 \times 10^6 \pm 2.63 \times 10^5$	0.14	
	Unidentified by-product II	$9.53 \times 10^5 \pm 2.96 \times 10^5$	0.08	
280	Sulfadimidine	$9.64 \times 10^8 \pm 4.03 \times 10^7$	98.87	1.13
	4,6-dimethyl-2-pyrimidinamine	$7.20 \times 10^6 \pm 7.61 \times 10^4$	0.74	
	Aniline	$2.46 \times 10^6 \pm 2.96 \times 10^5$	0.25	
	Unidentified by-product I	$1.07 \times 10^6 \pm 1.72 \times 10^5$	0.11	
	Unidentified by-product II	$2.98 \times 10^5 \pm 6.41 \times 10^5$	0.03	

Table S2: GC-MS signals from a 50 mM sulfadimidine standard and the related by-products resulting from the thermal decomposition of sulfadimidine within the GC injector, set at a split ratio of 1:10.

Injector temp. (°C)	Compound	Average peak area	Relative proportion (%)	Sum of by-products (%)
180	Sulfadimidine	$9.88 \times 10^8 \pm 1.50 \times 10^8$	100.00	
200	Sulfadimidine	$1.39 \times 10^9 \pm 4.85 \times 10^7$	100.00	
220	Sulfadimidine	$8.08 \times 10^8 \pm 3.77 \times 10^7$	100.00	
240	Sulfadimidine	$6.99 \times 10^8 \pm 2.72 \times 10^7$	99.74	0.26
	4,6-Dimethyl-2-pyrimidinamine	$1.81 \times 10^6 \pm 3.24 \times 10^5$	0.26	
260	Sulfadimidine	$6.37 \times 10^8 \pm 1.92 \times 10^7$	99.58	0.42
	4,6-Dimethyl-2-pyrimidinamine	$2.70 \times 10^6 \pm 2.31 \times 10^5$	0.42	
280	Sulfadimidine	$5.67 \times 10^8 \pm 2.77 \times 10^7$	98.90	1.10
	4,6-Dimethyl-2-pyrimidinamine	$3.07 \times 10^6 \pm 6.39 \times 10^5$	0.53	
	Unidentified by-product I	$2.48 \times 10^6 \pm 5.23 \times 10^5$	0.43	
	Unidentified by-product II	$7.91 \times 10^5 \pm 2.70 \times 10^4$	0.14	

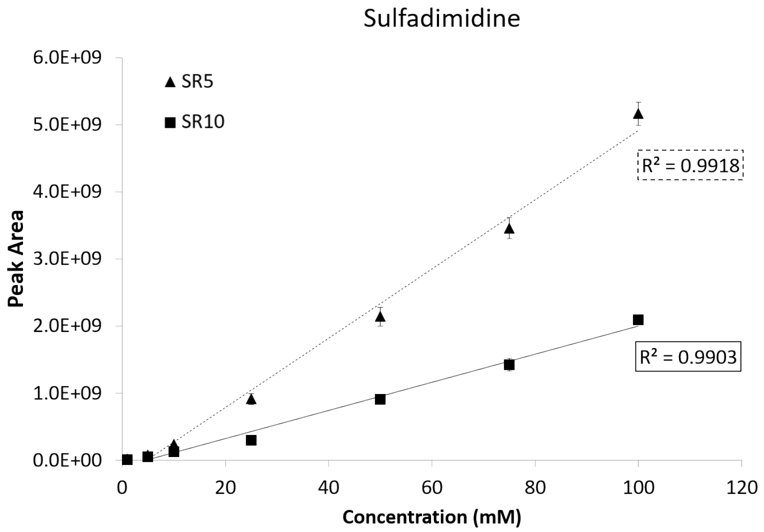

Figure S3: Peak areas of sulfadimidine at various concentrations on GC-MS. Triangles represent sulfadimidine standards injected at a split ratio of 1:5, whereas squares correspond to standards injected at a split ratio of 1:10.

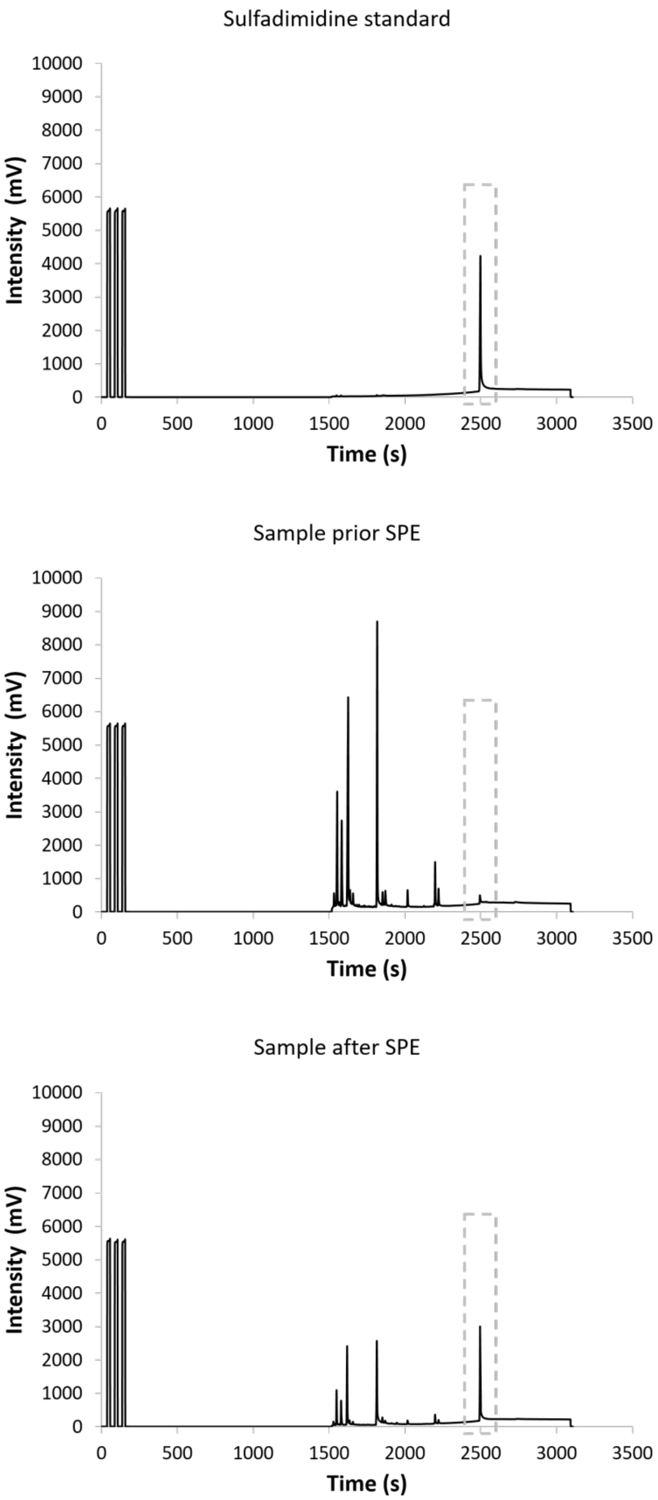

Figure S4: Chromatographic comparison illustrating the effectiveness of the SPE method in reducing matrix interferences, based on GC-IRMS analysis. The upper panel displays a 5 mM sulfadimidine standard (3 µL injection volume, split ratio 1:10), the middle panel shows a sulfadimidine-containing environmental sample prior to SPE treatment (5 µL injection volume, split ratio 1:5), and the lower panel presents the same sample after SPE treatment (3 µL injection

volume, split ratio 1:10). For better visibility, only the mass trace 44 is shown in the chromatograms. The peak of sulfadimidine, with a retention time of approximately 2500 s, is indicated by a gray dashed-line frame in all panels. The significant reduction in matrix-induced signals in the lower panel demonstrates the effectiveness of the SPE method in concentrating sulfadimidine while effectively reducing interferences.

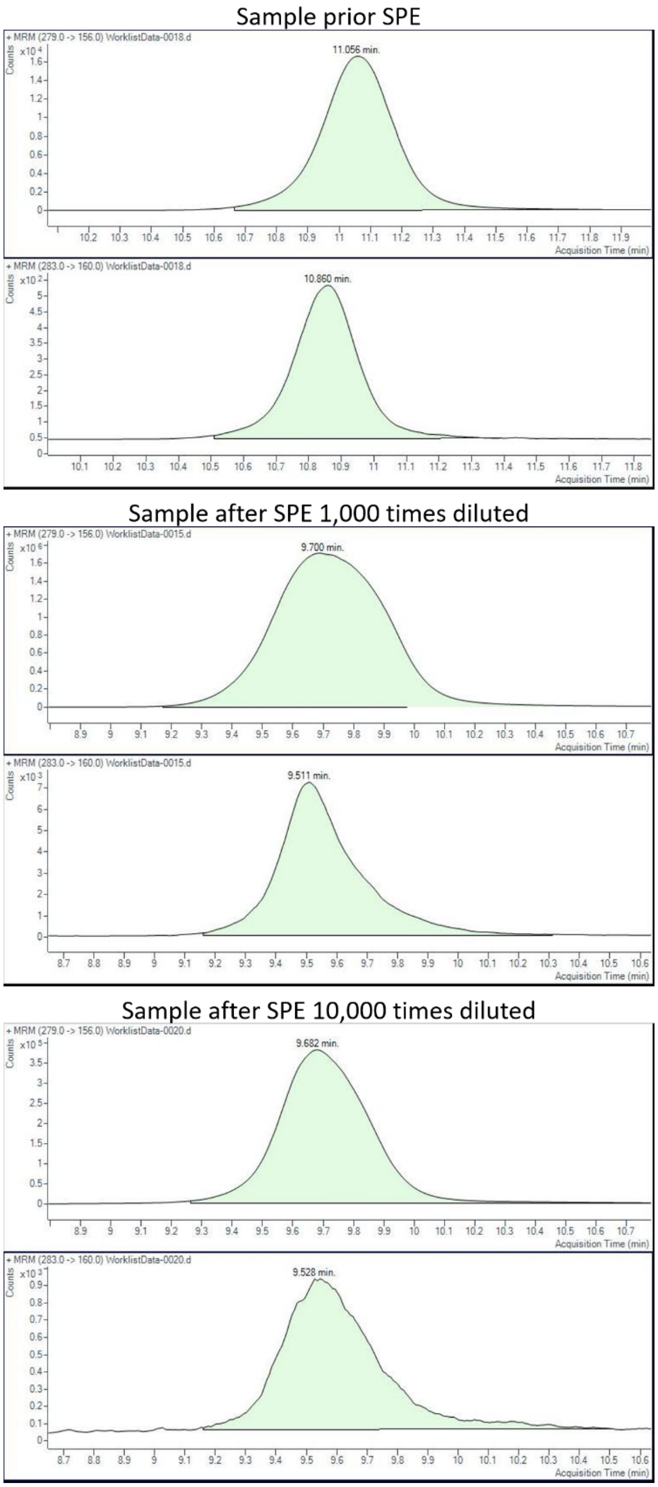

Figure S5: Chromatographic comparison of environmental samples before and after SPE treatment, demonstrating the effectiveness of the SPE method in concentrating sulfadimidine. The upper panel shows an environmental sample prior to SPE treatment, while the middle and lower panels display the same sample after SPE treatment, with 1,000-fold and 10,000-fold dilution, respectively. The results were obtained using High Performance Liquid Chromatography

– Triple Quadrupole Mass Spectrometry (HPLC–MS/MS) with dynamic Multiple Reaction Monitoring (dMRM). To ensure accurate quantification, sulfadimidine-d4 is used as an internal standard at a consistent concentration of 10 μg/L across all samples. Each panel is divided into two blocks: the upper block shows the mass transition and peak for sulfadimidine (m/z = 279 → 156), and the lower block represents the sulfadimidine-d4 internal standard (m/z = 283 → 160). While visually detecting matrix interference is challenging due to high dilution, potential matrix contributions are minimized through internal standard recovery and serial dilution during quantification.
